# Customized Titanium Mesh for Guided Bone Regeneration in the Posterior Mandible in a Patient Previously Treated with Bisphosphonates

**DOI:** 10.1155/2022/5174075

**Published:** 2022-10-22

**Authors:** Johannes Müller, Rüdiger Junker

**Affiliations:** ^1^Department of Prosthodontics and Biomaterials, Danube Private University, Krems, Austria; ^2^Deputy Medical Director, Danube Private University, Krems, Austria

## Abstract

We describe the restorative treatment of an 86-year-old female patient who was referred to our specialist prosthodontics clinic. Due to secondary osteoporosis, she underwent oral antiresorptive therapy with ibandronic acid for 10 years. Although she was classified as a patient at increased risk of drug-induced osteonecrosis of the jaw, she eventually signed a consent form for fixed implant-supported prosthetic rehabilitation in her 4th sextant. However, after tooth extraction, the bone ridge was too small at the intended implant positions. Therefore, guided bone regeneration was performed with a computer-aided design/computer-aided manufacturing three-dimensional printed titanium mesh. Finally, a continuous augmented hard tissue ridge above 7 mm was found, and three implants were inserted. Eventually, after four months of submerged healing, the implants were loaded with single crowns. Now, the patient is seen regularly for supportive peri-implant therapy.

## 1. Introduction

In our time, prosthetic rehabilitation of missing teeth with dental implants has become a predictable treatment option [1, 2]. However, a common clinical problem in implant dentistry is the lack of sufficient bone width and bone height to allow for prosthetically driven implant position and placement [3]. In view of this, scientific evidence supports lateral [3, 4] and vertical [3, 5] bone augmentation prior to implant placement as effective procedures to achieve sufficient ridge width and height for implant placement. In this context, guided bone regeneration (GBR)—in principle, the application of a barrier membrane to exclude cells from the soft tissue to allow protected migration of osteoblasts and eventually bone formation [6]—with resorbable, collagenous barrier membranes in combination with space maintaining xenografts is considered useful and predictable [3–5, 7]. However, such a combination might lack mechanical stability, which ultimately leads to a smaller increase in bone volume [4, 9]. To overcome this, individualized computer-aided design/computer-aided manufacturing (CAD/CAM) three-dimensional printed titanium meshes have been successfully introduced into GBR [10].

Here, we present the rehabilitation of the posterior mandible—in an elderly patient previously under bone resorptive therapy—with implants and implant-supported single crowns after guided bone augmentation supported by a printed titanium mesh.

## 2. Patient Information

**Figure 1:**
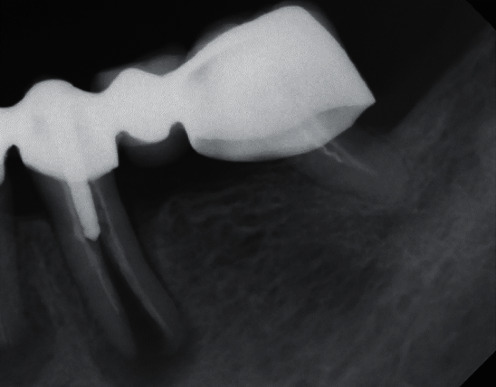
Radiograph tooth 35 at intake August 2018.

An 86-year-old female patient was referred to our specialist prosthodontic clinic in August 2018 for comprehensive therapy due to a vertical root fracture on tooth 35 (Figure 1) and the patient's desire for fixed rehabilitation. She presented with fixed, tooth- and implant-supported prosthetic restorations that were fitted about 17 years ago. But in recent years, she has not seen a dental hygienist or a general dentist regularly. She has also never smoked and is mentally and physically fit. However, due to secondary osteoporosis, she underwent oral antiresorptive therapy with ibandronic acid (i.e., one 150 mg tablet once a month; Bonviva®, Future Health Pharma GmbH, Wetzikon, Switzerland) from 2005 to 2015. For the treatment of osteoporosis, she continues to take Calciduran® vitamin D3 tablets (calcium carbonate; Recipharm Stockholm AB, Jordbro, Sweden) once a day and Oleovit® D3 drops (colecalciferol; Fresenius Kabi Austria GmbH, Graz, Austria) once a week. The patient is otherwise healthy. Her chief complaint was bite pain in the lower left posterior mandible. In addition, she insisted on fixed prosthodontic rehabilitation.

## 3. Clinical and Radiographic Findings

**Figure 2:**
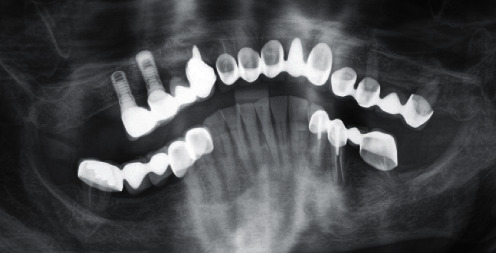
DPT section at intake August 2018.

**Figure 3:**
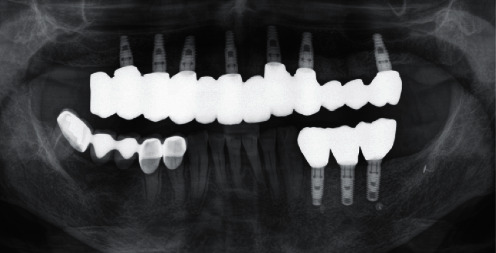
Section of a DPT immediately after cementation of the single crowns.

In brief, at intake, teeth 13, 12, 11, 21, 22, 23, 24, 25, and 27 as well as implants in regions 15 and 16 were present with corresponding fixed prostheses (Figure 2). Due to insufficient crown margins, caries, tooth mobility, and peri-implant bone loss, it was decided to extract all teeth in the maxilla and rehabilitate it with a one-piece fixed dental prosthesis (FDP) on seven implants (Table 1 and Figure 3). This treatment will not be presented and discussed in detail in this case report. In the mandible, teeth 37, 35, 34, 33, 32, 31, 41, 42, 43, 44, and 48 were present with corresponding fixed prostheses (Figure 2). Here, extractions of 37 (insufficient root canal treatment), 35 (vertical root fracture), and 34 (insufficient root canal treatment associated with peri-apical radiolucency), and replacement with three implant-borne single crowns were planned. No intervention was planned for the 6th sextant (Table 1). In addition, the patient had acceptable oral hygiene (Approximal Plaque Index 28%) but is prone to periodontitis (could be classified as stage III, grade B [11]).

## 4. Timeline

The timeline for the treatment of the left posterior mandible is shown in Table 2.

## 5. Therapeutic Intervention

As mentioned above, in the 4th sextant, the extractions of teeth 34 due to insufficient root canal treatment associated with peri-apical radiolucency, 35 due to a vertical root fracture, and 37 due to insufficient root canal treatment (as well as assuming that it would be unreasonable to treat and retain this tooth under the concept of prosthetic rehabilitation with implant-supported single crowns), and subsequent rehabilitation with three implant-supported single crowns were planned.

## 6. Risk Assessment for Medication-Related Osteonecrosis of the Jaw

Due to secondary osteoporosis, the patient had been taking oral antiresorptive medication (i.e., the bisphosphonate ibandronic acid, one 150 mg tablet once per month; Bonviva®, Future Health Pharma GmbH) from 2005 to 2015. Unfortunately, drug-induced osteonecrosis of the jaw (medication-related osteonecrosis of the jaw [MRONJ]) has been reported as a rare side effect of bisphosphonates. It is defined as exposed bone or bone that can be probed through an intraoral or extraoral fistula in the jaw region that has been present for more than eight weeks in patients who have been treated with anti-resorptive drugs in the past, without radiotherapy to the jaw or obvious metastatic disease of the jaw [12]. In this context, it should be understood that dentoalveolar surgery (e.g., GBR or placement of dental implants) is considered a risk factor for MRONJ [13]. However, the risk of MRONJ, for example, after the placement of dental implants, is unknown [14, 15]. However, as bisphosphonates are known to remain in the body for a considerable time after cessation of use, it is recommended that patients who have taken the drug for more than five years should be assigned to a risk group as if they were still taking the drug. [13]. Therefore, the patient was classified as a higher risk patient [13]. This risk assessment was discussed between the treating dentists and, of course, with the patient. Finally, the patient gave informed consent to the treatment (i.e., GBR and dental implants).

## 7. GBR—Planning

**Figure 4:**
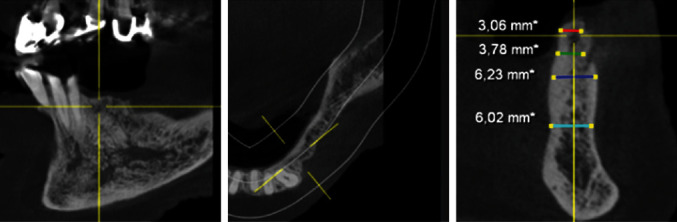
DVT—section region 34.

**Figure 5:**
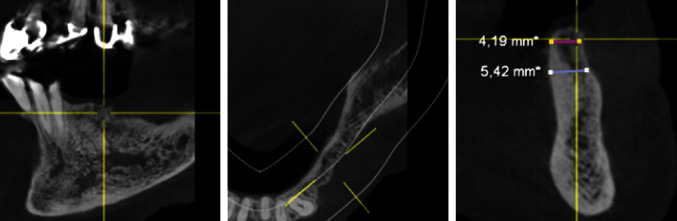
DVT—section region 35.

**Figure 6:**
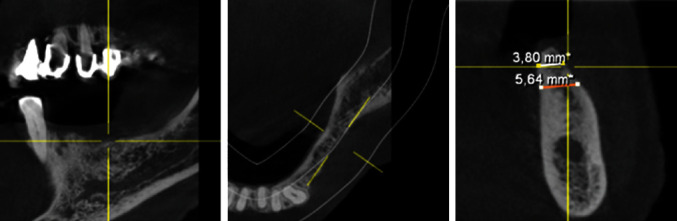
DVT—section region 36.

After the extraction of teeth 34, 35, and 37 and thirteen months of bone healing, a digital volume tomography (DVT) of the jaws was made in June 2020 for final treatment planning. Tapered Screw Vent TSVT MTX Zimmer Biomet™ implants (Zimmer Biomet Holdings Inc., Warsaw, IN, USA) were considered. The minimum diameter of this type of implants used for the replacement of premolars and molars is 3.7 mm, and the minimum length is 8 mm. When measuring the intended implant positions in regions 34, 35, and 36, the bone crest was found to be too narrow (<5 mm; Figures 4–6). Therefore, a GBR with a CAD/CAM-printed three-dimensional titanium mesh (YxOss CBR®; ReOss®, Filderstadt, Germany) in combination with autogenous bone (AB) and xenografts (Bio-Oss®/Bio-Gide®; Geistlich, Baden-Baden, Germany) was planned and prepared.

**Figure 7:**
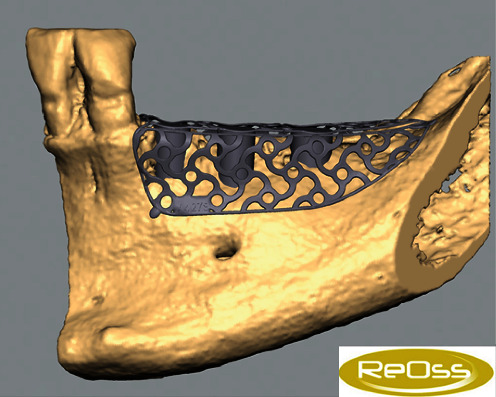
Individualized YxOss CBR® titanium mesh designed and visualized by ReOss®.

To create the conditions for successful implant placement and osteointegration, the first step was to simulate a primary horizontal augmentation to 8 mm in the 34–37 regions on the patient's DVT (Orthophos SL 3D, Dentsply Sirona Deutschland GmbH, Bensheim, Germany) from June 2020. The data were uploaded to and discussed with experienced clinical consultants (ReOss®, Filderstadt, Germany). Based on this, an individual YxOss CBR® titanium mesh was designed and visualized by ReOss® (Figure 7). After evaluation of the three-dimensional design and position by the treating specialist dentist, it was manufactured and delivered.

### 7.1. GBR—First Surgical Intervention

**Figure 8:**
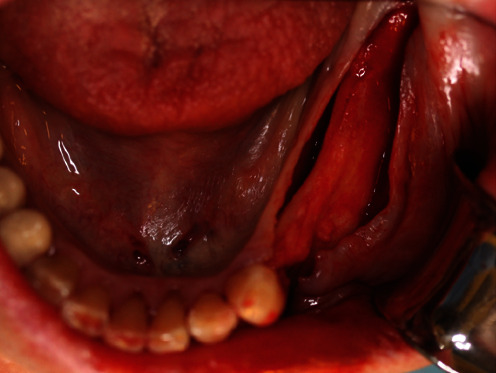
Muco-periosteal flap in the 4th sextant.

**Figure 9:**
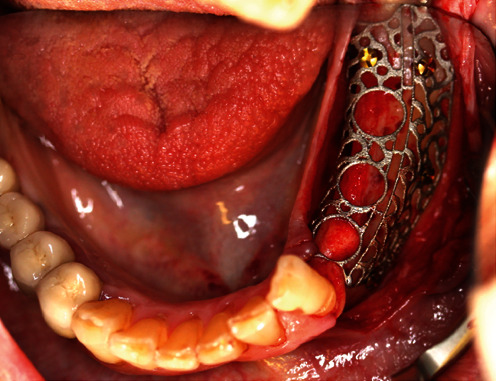
YxOss CBR® titanium mesh screwed onto the bone.

**Figure 10:**
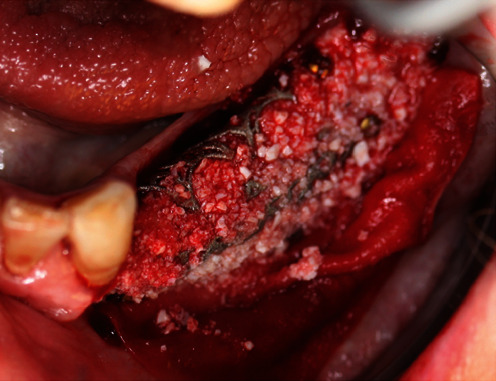
YxOss CBR® titanium mesh filled with the bone replacement graft.

**Figure 11:**
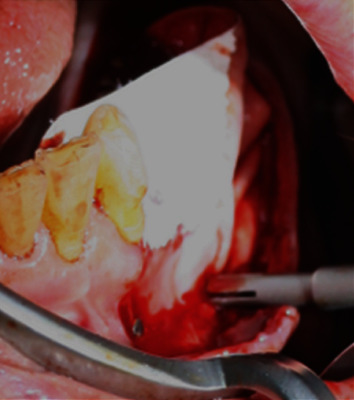
Bio-Gide® membrane stabilized in its position with titanium pins.

**Figure 12:**
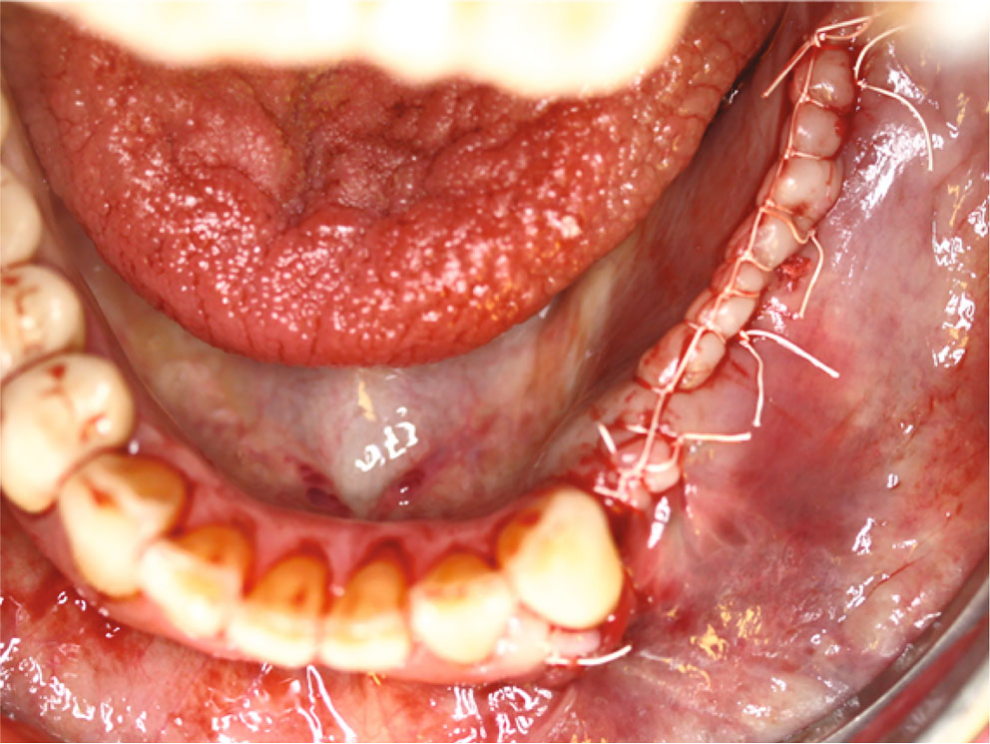
GORE-TEX® suture in place.

**Figure 13:**
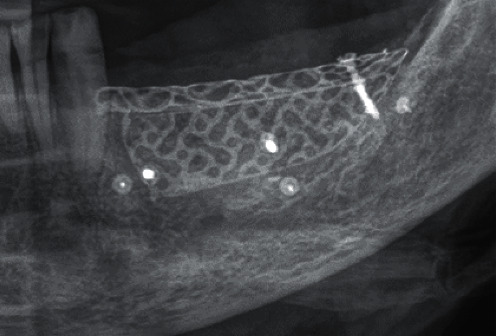
DPT section after GBR surgery was completed.

**Figure 14:**
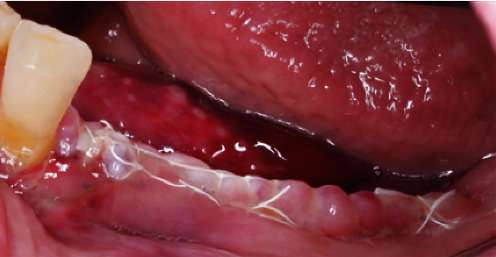
Day 1 after surgery.

**Figure 15:**
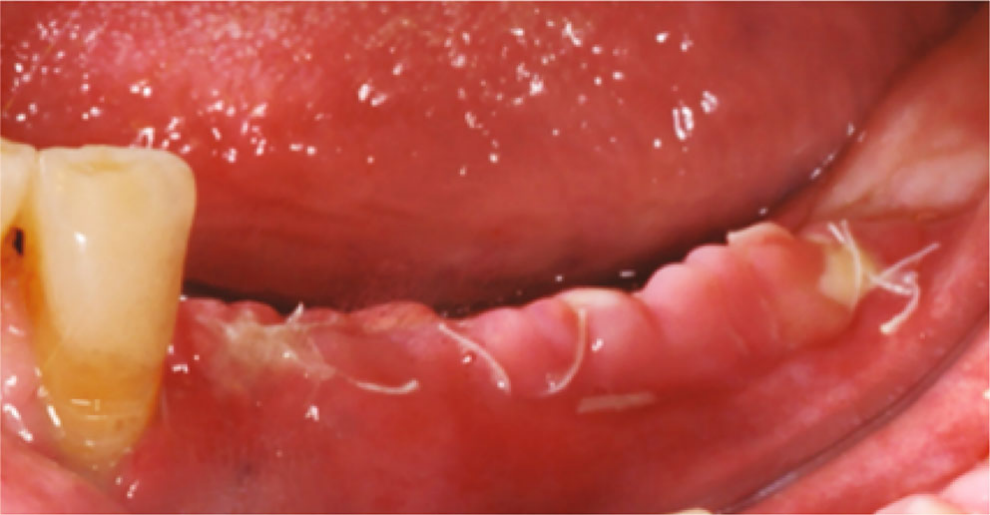
Day 7 after surgery.

On July 9, 2020, the corresponding surgical procedure was performed. As premedication, the patient received 40 mg Urbason® (methylprednisolone; Sanofi-Aventis Deutschland GmbH, Frankfurt, Germany), 1 g Augmentin® (amoxicillin + clavulanic acid; GlaxoSmithKline Pharma GmbH, Vienna, Austria), and 600 mg Ibuprofen® (nonsteroidal anti-inflammatory painkiller; BASF, Ludwigshafen, Germany) one hour before the surgery. Furthermore, regions 38–33 were anaesthetized with a total of 5.1 ml Ultracain® D-S forte (Sanofi-Aventis Deutschland GmbH, Frankfurt am Main, Germany). Then, after a mid-crestal incision form region 38 into the gingival sulcus of 33 and a realising incision into the vestibulum distal to 33, a muco-periosteal flap was prepared (Figure 8). Next, the sterilized YxOss CBR® titanium mesh was tried on and checked for accuracy of fit. Buccal cortical bone was then harvested apically and distally to the final mesh position for mixing with a xenograft (Safescraper™ Twist Cortical Bone Collector; Zimmer Biomet Austria GmbH, Vienna, Austria). Subsequently, to support adequate healing of the bone graft, the crestal and buccal cortical bone of the bed to be augmented was perforated with a drill (alveolar process drill: diameter 1.2 mm and length 8 mm; Straumann Holding AG, Basel, Switzerland). After repositioning the YxOss CBR® titanium mesh, holes were drilled through the corresponding mesh openings (alveolar process drill; Straumann Holding AG), and the mesh was screwed onto the bone with four titanium screws (titanium osteosynthesis screws: diameter 1.5 mm and length 8 mm; Straumann Holding AG; Figure 9). Then, 0.5 g Bio-Oss® small granules (0.25–1 mm) and 2 g Bio-Oss® large granules (1–2 mm) were mixed with autologous bone and blood from the surgical site and placed in the titanium mesh (Figure 10). Thereafter, a Bio-Gide® membrane (30mm × 40mm) was cut to cover the YxOss CBR® titanium mesh and about 3 mm of the surrounding bone in a single layer. The membrane was stabilized in position with three titanium pins (Straumann Holding AG; Figure 11). To ensure tension-free wound closure, underlying vertical mattress sutures with absorbable suture material (Vicryl 4-0; Ethicon, Johnson & Johnson Medical GmbH, Norderstedt, Germany) were placed. Primary wound closure was achieved with a continuous suture (GORE-TEX® Suture CV5; W.L Gore & Associates, Phoenix, AZ, USA). In addition, a sling suture was applied to 33 for coronal positioning and stabilisation of the mucoperiosteal flap (Figure 12). A digital pantomogram (DPT) was obtained immediately after suturing (Figure 13). Augmentin® (2 g × 1 g daily, for 7 days) and Ibuprofen® (3 mg × 600 mg daily, for 3 days, then as needed) were prescribed, and the patient was again informed about postoperative behaviour (e.g., cooling). An absence of at least three weeks from her interim prosthesis was agreed with the patient. In addition, the patient was asked not to brush the surgical area until the suture was removed, but to rinse regularly (at least twice a day) with Chlorhexamed® (0.1%; GlaxoSmithKline Consumer Healthcare GmbH, Munich, Germany) according to the instructions for use. From the first postoperative day until the removal of the sutures, the surgical area was checked and cleaned (cotton swabs soaked with Chlorhexamed® 0.1%) every other day (Figures 14 and 15). In addition, plaque on adjacent teeth was removed with curettes (Hu-Friedy Mfg. Co., LLC., Frankfurt, Germany). Until suture removal, twelve days after surgery, the wound healing was uneventful.

### 7.2. GBR—YxOss CBR® Titanium Mesh Removal

**Figure 16:**
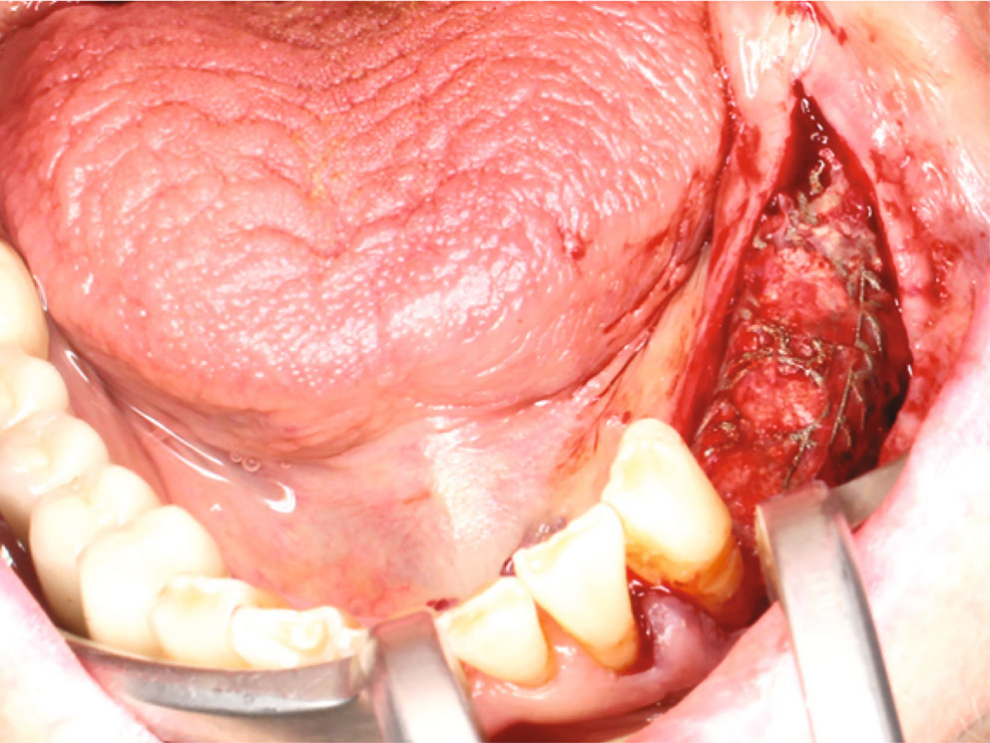
Exposed YxOss CBR® titanium mesh.

**Figure 17:**
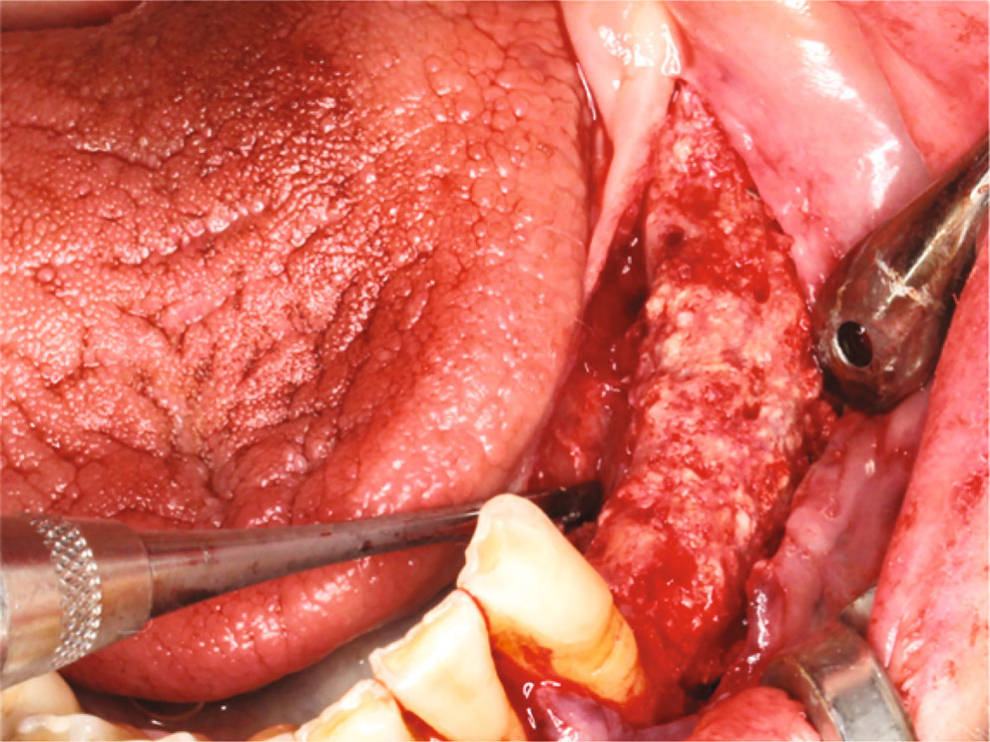
Ossified graft after YxOss CBR® titanium mesh removal.

After an uneventful—completely submerged—healing period of ten months, the titanium mesh was surgically removed on April 20, 2021 (delay due to COVID-19; usually 6 months according to Geistlich for BioOss®). As premedication, the patient received 1 g Augmentin® and 600 mg Ibuprofen® one hour before the surgery. In addition, regions 38–33 were anaesthetized with a total of 6.8 ml Ultracain® D-S forte. Then, after a mid-crestal incision from region 38 into the gingival sulcus of 33 (to keep the surgical area as small as possible without releasing incision), a muco-periosteal flap was prepared (Figure 16), and the YxOss CBR® titanium mesh, the four associated titanium screws, and one titanium pin were removed. Due to the decision to keep the surgical area as small as possible, the disto-apical and distal titanium pins were left in situ. Obviously, the graft was ossified (Figure 17).

### 7.3. Implant Placement

**Figure 18:**
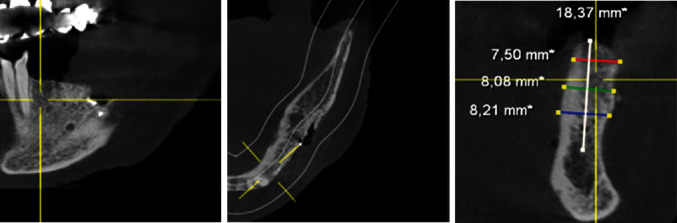
DVT—section; implant position region 34.

**Figure 19:**
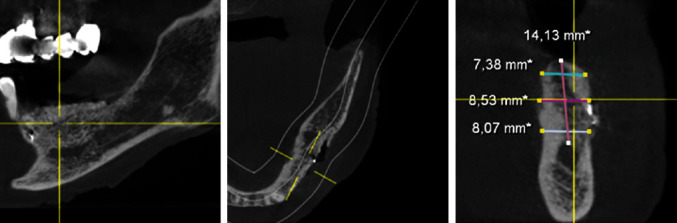
DVT—section; implant position region 35.

**Figure 20:**
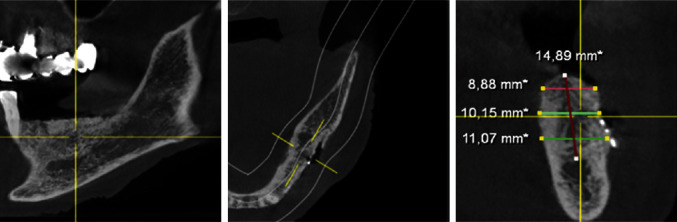
DVT—section; implant position region 36.

**Figure 21:**
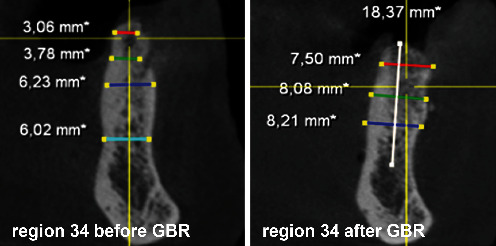
DVT—sections; implant position region 34 before and after GBR.

**Figure 22:**
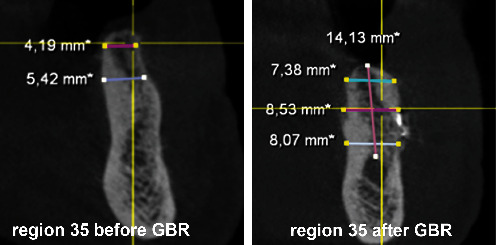
DVT—sections; implant position region 35 before and after GBR.

**Figure 23:**
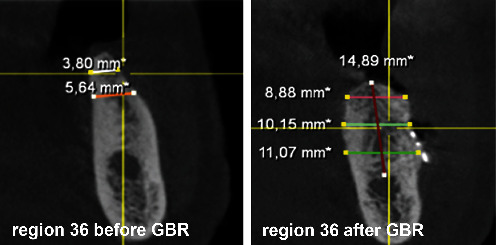
DVT—sections; implant position region 36 before and after GBR.

To plan the implant position, the bone edges were smoothed, and a DVT was taken immediately after removal of the YxOss CBR® titanium mesh. A continuous augmented hard tissue ridge, at the coronal margin above 7 mm, was found. Furthermore, the augmented hard tissue volume could be clearly distinguished from the native bone. As mentioned above, Tapered Screw Vent TSVT MTX Zimmer Biomet™ implants with a minimum coronal diameter of 3.7 mm and a minimum length of 8 mm had to be considered for the replacement of premolars and molars. When measuring the intended implant positions in regions 34, 35, and 36, the bone ridge was found to be sufficient in width and height (width: >7 mm, height (distance from coronal edge to upper edge of mandibular canal: >14 mm; Figures 18–20). Figures 21–23 show the approximate comparison of the hard tissue volume in the different regions where the implants were to be placed before and after GBR.

**Figure 24:**
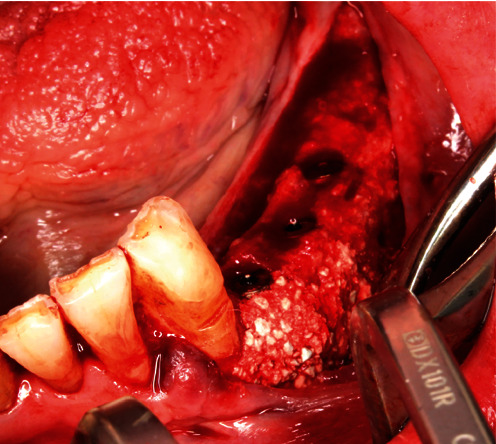
Tapered Screw Vent TSVT MTX Zimmer Biomet™ implants inserted in region 34, 35, and 36.

**Figure 25:**
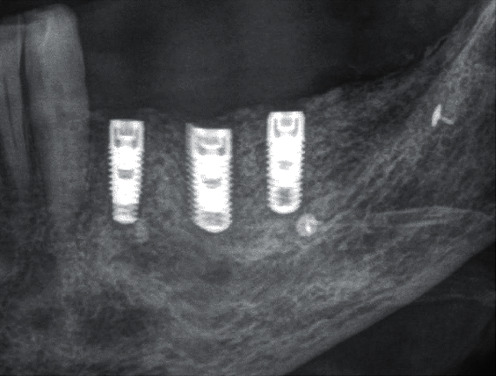
DPT—section; Tapered Screw Vent TSVT MTX Zimmer Biomet™ implants inserted in region 34, 35, and 36.

Immediately after planning the implant position, three Tapered Screw Vent TSVT MTX Zimmer Biomet™ implants (region 34: diameter 3.7 mm and length 10 mm; region 35: diameter 3.7 mm and length 10 mm; region 36: diameter 4.1 mm and length 10 mm) were implanted slightly subcrestal with an insertion torque of 35 Ncm each (Figure 24). Primary wound closure for healing of the submerged implants was achieved with interrupted single sutures (GORE-TEX® suture CV5). A DPT was taken immediately after suturing (Figure 25). Again, Augmentin® (2 g × 1 g daily, for 7 days) and Ibuprofen® (3 g × 600 mg daily, for 3 days, then as required) were prescribed, and the patient was asked not to brush the surgical area until the suture was removed, but to rinse regularly (at least twice a day) with 0.1% Chlorhexamed®. From the first postoperative day until the removal of the suture, the surgical area was checked and cleaned (cotton swabs soaked with Chlorhexamed® 0.1%) every fourth day. Plaque on the adjacent teeth was also removed with curettes. Until the suture was removed, thirteen days after surgery, wound healing was uneventful.

### 7.4. Prosthodontics and Supportive Therapy

In August 2021, after four months of submerged healing, the prosthetic phase was initiated. In detail, after surgical exposure of the implants and open impression technique (ImpregumTM PentaTM; 3 M Deutschland GmbH, Neuss, Germany), individual single crowns and corresponding abutments were finally designed (ExocadTM GmbH, Darmstadt, Germany). The individual abutments were milled from Zimmer Biomet™ pre-milled abutment blanks (Zfx™ GenTek™ Titanium Pre-milled Abutment Blank, 4.5 mm diameter). In addition, the matching single crowns were monolithically milled from a zirconia oxide blank (zircon GEN-X®; Amman Girrbach™, Koblach, Austria) and shaded according to the patient needs. Finally, the crowns were cemented onto the titanium abutments with KetacCem® (3MTM; Vienna, Austria). Immediately after cementation, a DPT was taken to check the exact fit and to exclude cement residues (Figure 3).

Thereafter, the patient received two further appointments, each two weeks apart, to (1) check and, if necessary, correct static and dynamic occlusion and (2) check and, if necessary, support oral hygiene. In the further course, the patient was enrolled in a supportive therapy programme, and visits were arranged every three months.

### 7.5. GBR—Calculated Hard Tissue Gain

**Figure 26:**
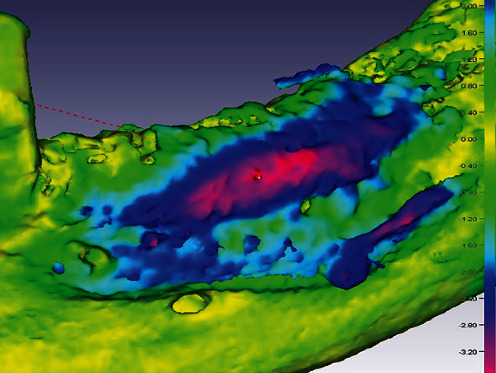
Graphical representation of the volume gain. The colour coding divides the gain from 0.0 mm (yellow) to 4.0 mm (red) at the respective positions of the third quadrant.

Figure 26 shows an overlay of DVTs before (2019) and after guided bone augmentation (2021). For this purpose, the corresponding 3D data sets were converted into 3D STL files using CoDiagnostiX® software (Dental Wings™, Montreal, QC, Canada) and then overlaid using Autodesk Netfabb 2020.0® software (Autodesk™, San Rafael, CA, USA). Furthermore, a matching was performed with the software Final Surface® (Gesellschaft zur Förderung angewandter Informatik e.V., GFaI, Berlin, Germany; modified best-fit method), and a volume gain of 1.3–1.4 cm^3^ was calculated. However, it should be clearly stated that this calculation method is not validated and that some referencing error (i.e., inaccuracies due to, e.g., artefact reduction during digital processing of the datasets used or conversion into STL files) remains in the 3D composite image.

## 8. Discussion

Here, we describe the restorative treatment of an 86-year-old patient who was referred to our specialist prosthodontics clinic in August 2018. Due to secondary osteoporosis, she received oral antiresorptive therapy with ibandronic acid from 2005 to 2015. Despite being classified as a patient at higher risk of MRONJ [13], she insisted on fixed implant-supported prosthetic rehabilitation in her 4th sextant and eventually signed the informed consent form. However, after extraction of teeth 34, 35, and 37 and thirteen months of bone healing, it was found that the coronal aspect of the bone ridge was too small (<5 mm horizontally) at the planned implant sites in regions 34, 35, and 36. Therefore, GBR was performed with a CAD/CAM-printed three-dimensional titanium mesh in combination with AB and a xenographic bone replacement graft. To create the conditions for successful implant placement and osseointegration, a horizontal augmentation of 8 mm ridge width in regions 34–37 was planned and performed. After ten months of submerged healing, a new DVT was taken, and a hard tissue ridge was found at the coronal margin, which was consistently above 7 mm. This allowed three implants (region 34: diameter 3.7 mm and length 10 mm; region 35: diameter 3.7 mm and length 10 mm; region 36: diameter 4.1 mm and length 10 mm) to be placed slightly subcrestal with an insertion torque of 35 Ncm each. After a four-month healing phase, the implants were finally restored with single crowns. Now the patient is seen regularly—every three months—for supportive peri-implant therapy.

During treatment planning, the previous antiresorptive therapy with Bonviva® (i.e., orally administered, 150 mg tablets once monthly; therapeutic indication: treatment of osteoporosis in postmenopausal women [16]) was critically reviewed. Each tablet of Bonviva® contains 150 mg ibandronic acid (molecular formula: C_9_H_23_NO_7_P_2_; structural formula: [1-hydroxy-3-(methylpentylamino)propylidene]diphosphonic acid), a medicine from the group of bisphosphonates for the treatment of, for example, osteoporosis. Hence, Bonviva® belongs to the pharmacological group of medicinal products for the treatment of bone diseases, bisphosphonates, and its Anatomical Therapeutic Chemical Classification System code is M05-BA06. Thereby, ibandronic acid is a highly effective bisphosphonate that belongs to the group of nitrogen-containing bisphosphonates (N-BPs; group of so called powerful antiresorptives [pARs]) that act selectively on bone tissue and specifically inhibit osteoclast activity without directly affecting bone formation. It does not interfere with osteoclast recruitment. Consequently, it leads to a progressive net increase in bone mass due to the reduction in increased bone turnover to premenopausal levels in postmenopausal women [16]. The principle molecular mechanisms underlying the inhibition of bone resorption by osteoclasts mediated by ibandronic acid are known. In brief, since bisphosphonates target bone mineral and osteoclasts are capable of releasing bone-bound bisphosphonate, a direct effect on mature osteoclasts appears to be the most important route of action. Ibandronic acid seems to act as analogues of isoprenoid diphosphate lipids, thereby inhibiting farnesyl diphosphate synthase. Inhibition of this enzyme in osteoclasts prevents the biosynthesis of isoprenoid lipids (farnesyl pyrophosphate and geranylgeranyl pyrophosphate), which are essential for posttranslational farnesylation and geranylgeranylation of small GTPase signalling proteins. Then, the loss of bone resorptive activity and osteoclast apoptosis is primarily a consequence of the loss of these geranylgeranylated small GTPases [17].

Unfavourably, MRONJ is a potentially severe adverse event affecting patients with osteoporosis (this applies also to patients with cancer, of course) who have been treated with, for example, N-BPs, pARs [18] as ibandronic acid. Thus, the European Public Assessment Report of the European Medicines Agency for Bonviva® lists osteonecrosis of the jaw (ONJ; i.e., MRNOJ) as a very rare adverse reaction (<1/10,000) reported in post-marketing settings in postmenopausal women receiving Bonviva 150 mg once monthly [16]. However, it should not be left unmentioned that this is generalized in a footnote to “Cases of osteonecrosis of the jaw have been reported, predominantly in cancer patients treated with medicinal products that inhibit bone resorption, such as ibandronic acid. Cases of ONJ have been reported in the post-marketing setting for ibandronic acid.” [16] And thus, also for Bonviva®, indicating, and consistent with published data, that pAR-related MRONJ has similar general MRONJ pathophysiology in cancer and osteoporosis patients [12, 19, 20], but that pAR-related MRONJ occurs less frequently in patients treated for osteoporosis (incidence of 0.01–0.03%) than in cancer patients (incidence of 1.8–5%) [12, 19, 21, 22]. Accordingly, that pAR-treated patients are at risk for MRONJ is reflected in guidelines [13, 23]. On this basis, we primarily classified our patient as being at higher risk of developing MRONJ. However, preclinical and clinical data suggest that most MRONJ cases require systemic risk factors (e.g., pARs) in combination with local oral risk factors, including inflammatory dental disease (e.g., periodontitis), tooth extractions, trauma from removable dentures, and possibly placement of dental implants [12, 19, 20, 24–39]. Be that as it may, even when looking very cautiously at scientific papers published to date, there seems to be convincing evidence that placing dental implants in patients receiving oral bisphosphonate therapy (as opposed to intravenous bisphosphonate therapy) has no significant negative impact on the development of MRONJ, implant survival, and implant success rate alone [15, 39–44]. Furthermore, since the teeth 34–37 would have had to be extracted anyway and periodontitis was successfully treated in advance and, moreover, as the general pathophysiology of MRONJ associated with pAR is at least similar in cancer and osteoporosis patients, and therefore trauma from removable dentures could be a theoretical risk factor for the development of MRONJ in patients with oral pAR therapy [35, 38], and since the patient presented here was otherwise healthy (i.e., beside an age of more than 60 years and female sex, no other non-dental risk factors for developing MRONJ; for review see Wan et al. [24]), we finally categorised her as a patient at lower risk for the development of MRONJ and were willing to realize the patient's request for a fixed—implant-supported—prosthetic restoration. However, the actual statistical or even individual risk of developing MRONJ (during or) after oral therapy for postmenopausal osteoporosis with Bonviva® following GBR and dental implantation remains unknown. All this was discussed with the patient in detail. Finally, the patient agreed to the therapy and signed the corresponding consent form. It should not go unmentioned that the treatment outcome confirms our assessment regarding not developing MRONJ.

Since oral surgical procedures should generally be considered risk factors for the development of MRONJ also in patients on oral pAR therapy (e.g., tooth extraction, bone augmentation, and implant placement), we chose a multistage approach. First, although the evidence especially for oral pAR therapy should be regarded as low, the teeth 34, 35, and 37 were extracted under a preventive protocol [28, 45–48]. In brief, before extraction, care was taken (especially in the 3rd quadrant) to ensure that plaque was reduced as much as possible and that there were no clinical signs of gingivitis [28]. As premedication, the patient received 1 g Augmentin® and 600 mg Ibuprofen® one hour before the intervention. In addition, immediately before tooth extraction, the oral cavity was thoroughly rinsed with Chlorhexamed® FORTE 0.2% for one minute. Furthermore, tension-relieving incisions were made for tooth extraction, bone edges were removed after the extraction, and the entire wound was completely closed in a passive manner (i.e., tension-free) [28, 46, 47]. In addition, Augmentin® (2 g × 1 g daily, for 7 days) and Ibuprofen® (3 mg × 600 mg daily, for 3 days, then as required) were prescribed. Furthermore, the patient was asked not to brush the surgical area until suture removal, but to rinse regularly (at least twice a day) with Chlorhexamed® 0.1%. Starting on the first post-surgical day until suture removal, the surgical area was checked and cleaned (cotton swabs soaked in Chlorhexamed® 0.1%) every second day. In addition, plaque on adjacent teeth was removed with curettes (Hu-Friedy Mfg. Co., LLC.). Until suture removal, wound healing was uneventful. Based on this experience, this prevention protocol was used accordingly for bone augmentation and implantation.

In general (i.e., not for patients with previous or current oral pAR therapy), the available evidence suggests that predictable correction of horizontal bone deficits that allow implant placement is possible through bone augmentation procedures (for review, see Naenni et al. [4]). In this systematic review and meta-analysis, various methods (but not graft-stabilising techniques with, e.g., a titanium mesh) and materials for lateral bone augmentation were analysed. The mean horizontal hard tissue gain was calculated to be 3.30 mm (95% confidence interval (CI): 2.81–3.79 mm; prediction interval (PI): 0.96–5.65 mm) for all available studies (i.e., twenty studies) and to 3.43 mm (95% CI: 2.83–4.03 mm; PI: 1.31–5.55 mm) for only seven studies that presented data for all three time points investigated (pre-augmentation, post-augmentation, and re-entry). Furthermore, the mean decrease of the augmented horizontal hard tissue was calculated as −1.33 mm (95% CI: −1.78 to −0.88 mm; PI: −2.98 to 0.32 mm (all studies) and −1.54 mm (95% CI: −2.17 to −0.91 mm; PI: −3.77 to 0.69 mm (seven studies with data for all three time points). This means, however, that due to the high variability of hard tissue augmentation achieved, predictability for actual treatment (for not graft-stabilising techniques with, e.g., a titanium mesh) is certainly difficult.

Moreover, for Bonviva® in particular, there was (and still is to our knowledge) no published evidence regarding bone augmentation procedures. For that reason, during treatment planning, it was assumed that since Bonviva® does not interfere with osteoclast recruitment and leads to an increase in bone mass [16], new bone formation should not be compromised during bone augmentation (i.e., GBR), and therefore, correction of the horizontal bone deficit should be predictably possible in the current patient to allow implant placement. Since, as mentioned above, any oral surgery should be considered a risk factor for the development of MRONJ, even in patients receiving oral pAR therapy, defect augmentation was not performed with bone harvested intraorally from a second surgical site (chin, ramus mandibulae), but for mixing with a xenograft, buccal cortical bone was collected apical and distal to the final mesh position with a Safescraper™.

For the augmentation method in the present case (i.e., Bio-Oss® mixed with AB, and Bio-Gide®; but not stabilized with a titanium mesh), there is some evidence regarding lateral hard tissue gain, graft reduction during wound healing, and predictability of implant placement [49]. In brief, in a randomized clinical trial (RCT) Bio-Oss®, particles were mixed by weight with AB particles either in a ratio of 90 : 10 (mean; 1.2 g Bio-Oss® : 0.14 g AB) or in a ratio of 60 : 40 (mean; 1.1 g Bio-Oss® : 0.7 g AB) together with autogenous blood from the surgical site. However, in contrast to the present case, 0.25 ml of fibrin glue was added to each of the graft mixtures to make the graft moldable and prevent the particles from migrating from the augmented area. Finally, all grafts were covered by Bio-Gide® membranes without further fixation. The width of the alveolar ridge ranged preoperatively from 1.6 to 6.1 mm and 1.4 to 6.5 mm, 3 and 6 mm from the top of the alveolar ridge, respectively. After a submerged healing period of approximately 7.5 months, the hard tissue gain was 0.4–6.3 mm (graft reduction: 0–94.9%) and 0.4–6.3 mm (graft reduction: −8.6% to 89.7%), 3 and 6 mm from the upper edge of the alveolar ridge, respectively. Finally, all the implants could be placed in all the planned locations, except for one, where the ridge was still too narrow, and the graft was very soft at this point. As noted for the review and meta-analysis by Naenni et al. [4], this again means that due to the high variability of hard tissue gain and graft reduction, predictable treatment planning (i.e., predictable implant placement) with the method and material used may indeed be difficult. In addition, Meijndert et al. [50–52] studied Bio-Oss® in combination with Bio-Gide® in one group (five patients) of a parallel-group RCT. In brief, all patients had a single-tooth diastema in the anterior region of the maxilla. Bio-Oss® was mixed with blood from the surgical site (but, unlike the present case, not mixed with AB, and no graft stabilising titanium mesh was used) and covered with a Bio-Gide® membrane. After six months of submerged healing, ridge enlargement of 2–3 mm was reported [50]. Two implants inserted in a Bio-Oss®/Bio-Gide® graft failed to osseointegrate and were lost within six months of placement [52]. In addition, the marginal peri-implant bone loss between 1 and 120 months after prosthetic loading (i.e., crown placement) was calculated to be −0.64 mm (SD: 1.46 mm) at mesial and −0.37 mm (SD: 1.47 mm) at distal sites in the Bio-Oss®/Bio-Gide® group. During this 10-year period, no further implant in the Bio-Oss®/Bio-Gide® group was lost [52]. But again, predictable treatment planning for long-term implant success may be difficult with the method and material used.

Therefore, to augment the region 34–37 predictably for successful implantation and osseointegration, a graft-stabilising three-dimensional printed titanium mesh for horizontal bone augmentation of up to 8 mm was planned and used in the present case. The graft biomaterials used were Bio-Oss® (mixed with AB and blood from the surgical site) and a Bio-Gide® membrane (to prevent ingrowth of connective tissue and to ensure osteogenic potential in the marginal area of the graft), stabilized with titanium pins. After a submerged healing period of ten months, a continuous augmented hard tissue ridge, at the coronal margin above 7 mm, was found, and all three implants could be placed in a prosthodontically driven position with an insertion torque of 35 Ncm each. Furthermore, after four months of submerged implant healing, the implants were clinically stable (i.e., osseointegrated) and restored with single crowns. Thus, the lateral bone width gain in the entire augmentation area in the 4th sextant was approximately between more than 3 mm and more than 5 mm. This is within the 95% prediction interval of bone width gain of the systematic review and meta-analysis on lateral bone augmentation before implant placement without graft stabilising techniques (e.g., a titanium mesh) published by Naenni et al. [4] and within the range of bone width gain (without graft stabilising technique) in the study by Mordenfeld et al. [49]. Moreover, such a continuous increase in hard tissue was never observed in our hands in similar GBRs without titanium mesh and might be attributed to the positional stability of the augmentation, which allows undisturbed bone healing free from compressive loads and other physical influences. Furthermore, compared to the data of Naenni et al. [4] and Mordenfeld et al. [49], the associated graft reduction was minimal in the current case.

Besides, there is a limited evidence for lateral bone augmentation with a graft-stabilising titanium mesh in general (for review see [53, 54]) and for the YxOss titanium mesh in combination with Bio-Oss® mixed with AB and Bio-Gide® in particular [55, 56] for lateral hard tissue gain, wound healing, and predictability of implant placement. In brief, Sagheb et al. [55] retrospectively analysed GBR (i.e., Bio-Oss® mixed with AB (AB form different intraoral donor sites and from the iliac crest or AB alone/YxOss titanium mesh/Bio-Gide® plus platelet-rich fibrin (PRF) membrane or Bio-Gide® alone) in 17 patients with 21 principally three-dimensional bone defects (i.e., vertical and horizontal bone augmentation). The postoperative healing was uneventful in 14 GBR sites (67%) during the follow-up time of six months until re-entry. However, at seven GBR sites (33%), exposure of the YxOss titanium mesh was observed after periods of five to twelve weeks after the first-stage surgery. Re-entry with explantation of the YxOss titanium mesh and simultaneous implantation of 44 implants was performed after six months. Cone beam computed tomography was performed preoperatively and six months postoperatively to measure bone height and width. A mean vertical bone gain of 6.5 mm (SD: 1.7 mm) and a mean horizontal bone gain of 5.5 mm (SD: 1.9 mm) were observed. Furthermore, after a mean follow-up time of 12 months (SD: 6 months) after second-stage surgery, none of the 44 inserted implants were lost, which corresponds to a survival rate of 100%. In the end, the authors only very generally concluded that individualized CAD-CAM fabricated titanium meshes are a safe and predictable procedure for large vertical and horizontal ridge augmentations. Similar results are reported by Hartmann and Seiler [56]. They also retrospectively analysed GBR (i.e., Bio-Oss® mixed with AB (in one patient, only Bio-Oss®; AB from different intraoral donor sites and from the iliac crest; ratio 1 : 1/YxOss titanium mesh/Bio-Gide® or Bio-Gide® plus an Advanced- (A-PRF®) membrane) in 55 patients with 68 three-dimensional bone defects (i.e., vertical and horizontal bone augmentation). Overall (i.e., Bio-Gide® or Bio-Gide® plus A-PRF® membrane), exposure of the YxOss titanium mesh was observed in 25% of treated defect sites (*n* = 17) in association with partial (11.8%, *n* = 8) and complete (15%, *n* = 1) loss of graft material. The YxOss titanium meshes were removed after a mean healing time of 6.5 months (SD: 2.7 months). A total of 98 implants were placed as planned. These authors also very generally concluded that the surgical protocol of individual bone regeneration has proven to be a promising technique for complex bone reconstructions. But the variability of GBR treatment performed in the studies of Sagheb et al. [55] and Hartmann and Seiler [56] (vertical and horizontal bone augmentation/different biomaterials) makes an actual evidence-based, meaningful comparison with the case presented here difficult.

Nevertheless, this case shows that GBR with a CAD/CAM-printed three-dimensional titanium mesh can predictably fulfil the desire for fixed, implant-supported single crowns, and a corresponding quality of life even in an older, medically compromised patient.

However, this result should not be generalized (predictable horizontal bone augmentation width, minimum reduction of the graft) as such a statement would require clinical trials with appropriate patient numbers.

## 9. Patient Perspective

To be able to describe an objective assessment of patient satisfaction, the Oral Health Quality questionnaire with 53 questions was used for the evaluation. There was a clear increase in the quality of life, and in particular, the patient did not describe any problems or complaints with chewing functionality or phonetics.

## Figures and Tables

**Table 1:** DPT, dental status at intake, and treatment planning.

Treatment plan		FDP														
			I		I	I		I		I	I			I		
			X	X		X	X	X	X	X	X	X	X		X	
Dental status			**FDP**				**FDP**					**FDP**				
	**M**	**M**	**I**	**I**	**M**									**M**		**M**
	**8**	**7**	**6**	**5**	**4**	**3**	**2**	**1**	**1**	**2**	**3**	**4**	**5**	**6**	**7**	**8**
	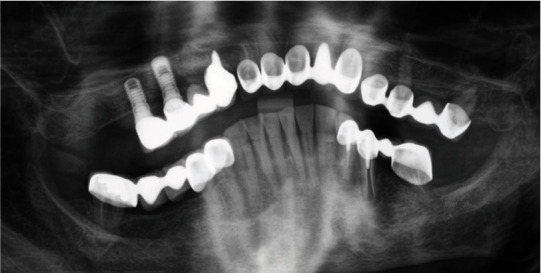															
Dental status	**8**	**7**	**6**	**5**	**4**	**3**	**2**	**1**	**1**	**2**	**3**	**4**	**5**	**6**	**7**	**8**
		**M**	**M**											**M**		**M**
	**FDP**											**FDP**				
Treatment plan												**I**	**I**	**I**		
												**SC**	**SC**	**SC**		

M: Missing tooth. I: Implant. X: To be extracted or explanted. SC: Single crown. FDP: Fixed dental prosthesis.

**Table 2:** Chronological order of the treatment.

Timeline—left posterior mandible	
Intake	August 2018
Clinical examination	
DPT	
Articulated models of maxilla and mandible	
Preliminary treatment plan and initial treatment	Up to June 2020
Oral hygiene improvement	
Non-surgical periodontal therapy	
Extraction of teeth 34, 35, and 37	
Supportive therapy 4 times per year	
DVT	June 2020
Definitive treatment plan	
Implant positions	
CAD-titanium mesh for guided bone regeneration	
First surgical approach (i.e., GBR)	July 2020
CAM-printed titanium mesh (YxOss®; ReOss, Filderstadt, Germany)	
Xenografts (Bio-Oss®/Bio-Gide®; Geistlich, Baden-Baden, Germany)	
Submerged healing	
Second surgical approach	May 2021
Removal of the titanium mesh	
Implant placement (Tapered Screw Vent Zimmer Biomet™; Zimmer Biomet Holdings Inc., IN, USA)	
Submerged healing	
Third surgical approach	August 2021
Uncovering of the implants	
Healing abutments	
Prosthodontics (i.e., loading the implants)	
Individualized titanium abutments	
Monolithic, coloured SCs (Zirkon GEN-X®; Amman Girrbach™, Koblach, Austria)	
Cemented (KetacCem®; 3M; Vienna, Austria)	

DPT: digital panoramic tomography. DVT: digital volume tomography. GBR: guided bone regeneration. CAD: computer assisted design. CAM: computer assisted manufacturing. SC: single crown.

## Data Availability

They are patient data. They are stored in our institution (Danube Private University's outpatient clinic in Krems, Austria) and are not publicly accessible.
